# *In Vitro* biomechanical study of meniscal properties in patients with severe knee osteoarthritis

**DOI:** 10.3389/fbioe.2025.1637649

**Published:** 2025-11-10

**Authors:** Yuqi Liu, Songhua Yan, Haixia Zhang, Jizhou Zeng, Kuan Zhang

**Affiliations:** 1 School of Biomedical Engineering, Capital Medical University, Beijing, China; 2 Department of Bone and Joint Surgery, Luhe Hospital, Capital Medical University, Beijing, China

**Keywords:** knee osteoarthritis, meniscus, tensile modulus, compressive modulus, inversefinite element analysis

## Abstract

**Introduction:**

The meniscus is a crucial component of the human knee joint, contributing to load transmission, stability, and lubrication. Meniscal injury can disrupt knee biomechanics and eventually lead to knee osteoarthritis (KOA). Quantifying the biomechanical properties of the meniscus is essential for understanding its role in knee joint function and pathology.

**Methods:**

This study aimed to determine the biomechanical properties of the meniscus in patients with severe KOA using experimental mechanical testing and an inverse finite element analysis (iFEA) model. Meniscal samples were collected and graded from patients undergoing total knee arthroplasty. Tensile (n = 113) and compressive (n = 137) tests were performed to obtain experimental data. An iFEA model with a Mooney–Rivlin hyperelastic formulation was developed to estimate the constitutive parameters of the meniscus through optimization algorithms.

**Results:**

In patients with severe KOA, the average tensile modulus of the lateral meniscus (57.9 ± 34.1 MPa) was approximately 40.5% higher than that of the medial meniscus (41.2 ± 28.8 MPa). At 10% strain, the average compressive modulus was 2.2 ± 1.5 MPa for the medial and 2.5 ± 1.5 MPa for the lateral meniscus. Compared with the 10% strain level, the compressive modulus of the medial and lateral menisci increased by 54.5% and 136% at 20% strain, and by 222.7% and 288% at 30% strain, respectively. Both tensile and compressive moduli exhibited a stepwise decrease with increasing degeneration. The iFEA model showed an excellent fit to the experimental data (R^2^ > 0.9).

**Discussion:**

The biomechanical properties of the meniscus in severe KOA patients differ substantially from those reported in healthy tissues, highlighting the need for caution when using literature-derived parameters in computational modeling. The iFEA framework provides a robust approach for predicting constitutive parameters across different degeneration levels and meniscal regions, offering valuable insights for personalized knee joint modeling and simulation.

## 1 Introduction

KOA is a prevalent chronic joint disease, with an increasing global incidence ranging from approximately 3.8%–17.6% (Dong et al., 2023; Giorgino et al., 2023; Michael et al., 2010; Mahmoudian et al., 2021). As the global population ages, these numbers are expected to continue to rise.

The menisci provide joint stability, load transfer, impact absorption, and knee lubrication (Sweigart et al., 2004; Vaienti et al., 2017; Walker and Erkman, 1975; Karahan et al., 2010; Saygi et al., 2005; Fukubayashi and Kurosawa, 1980). During daily activities, the meniscus is subjected to various forces such as tension, compression, and shear forces. Biomechanical testing is usually performed to quantify the effects of these forces on the meniscus tissue (Markes et al., 2020; Morejon et al., 2023; Maritz et al., 2021). Biomechanical characterization of the joint can accurately show the distribution of forces in the knee joint during various activities, and can identify abnormal stress concentrations. This can help to explain the mechanical causes of pain generation in patients with KOA, thus providing assistance in disease prevention and clinical treatment.

While extensive research has focused on the biomechanical properties of the meniscus in healthy individuals (Abraham et al., 2011a; Sandmann et al., 2009; Moyer et al., 2013; Martin Seitz et al., 2013), studies on the meniscus in patients with KOA remain limited. The main consensus is that meniscal degeneration affects compressive properties, leading to reduced tissue stiffness (Andrews et al., 2014; Katsuragawa et al., 2010; Sandmann et al., 2013; Son et al., 2013; Fischenich et al., 2015). Warnecke et al. (2020) found a gradual but not statistically significant decrease in tensile modulus as the degree of degeneration of the lateral meniscus progressed, while (Fischenich et al., 2015) found no significant change in tensile modulus as the meniscus degenerated. There were limited sample sizes in those studies, making definitive conclusions difficult as it is understandable that samples are indeed not easy to access.

Finite element analysis (FEA) is widely used in knee joint biomechanics to approximate the solution of boundary value problems in partial differential equations (Park et al., 2019; Chen et al., 2023). Currently, when performing FEA of the knee joint, most studies consider the meniscus as a linearly elastic material with a modulus of elasticity of 59 MPa and a Poisson’s ratio of 0.49 (LeRoux and Setton, 2002), while some literature set the meniscus as a linearly elastic and transversely isotropic material with a circumferential modulus of 120 MPa, the cross-section modulus of 5 MPa and a Poisson’s ratio of 0.3 (Park et al., 2019; Daszkiewicz and Łuczkiewicz, 2021). However, the meniscus is a nonlinear material because of its nonlinear stress-strain relationship and strain rate dependence.

Traditional mechanical testing often requires complex instrumentation and a large number of experimental samples to obtain more accurate material parameters. It is much more difficult, even impossible to obtain the biomechanical properties of the meniscus through mechanical testing most of the time. The iFEA has been widely used to determine the mechanical properties of materials (Korhonen et al., 2002; Lei and Szeri, 2007; Gao et al., 2023). Seyfi et al. (2018) optimized the intrinsic parameters of the bovine meniscus using the indentation test and iFEA. Freutel et al. (Freutel et al., 2015) analyzed the biomechanical properties of circumferential fibers of porcine meniscus using the iFEA. De Rosa et al. (2022) employed iFEM to successfully characterize the mechanical properties of collagen fibers within the meniscus, providing deeper insight into tissue-level mechanics. However, most existing simulations of KOA, including finite element analyses, still rely on the mechanical properties of healthy meniscal tissue, which may not accurately reflect the altered biomechanical environment in KOA patients. To address this issue, the present study aims to characterize the biomechanical properties of menisci from patients with severe KOA using both mechanical testing and iFEA.

## 2 Methods

### 2.1 Sample acquisition

**FIGURE 1:**
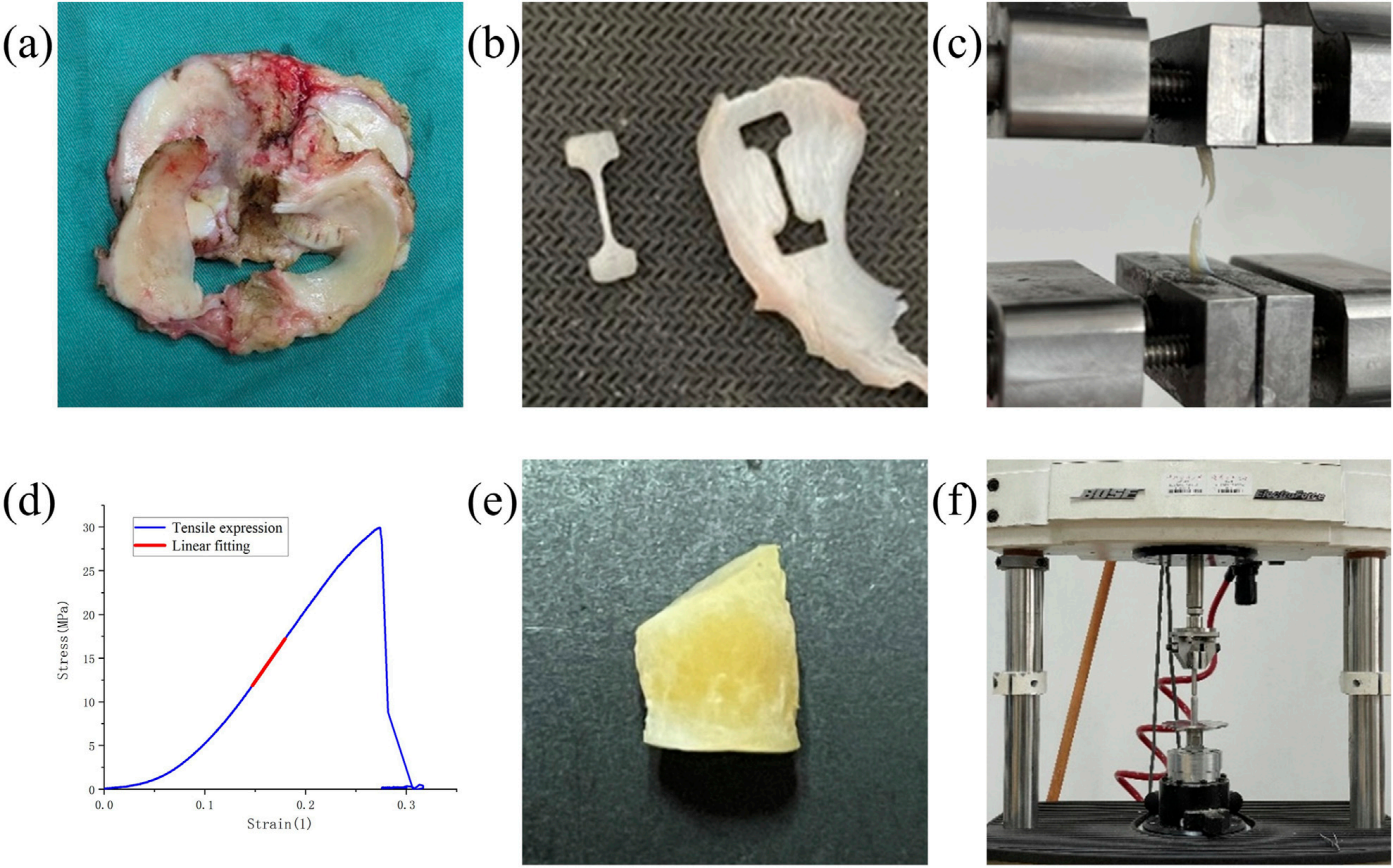
Experimental setup and result representation for meniscus biomechanical tests. **(a)** Meniscus in patients undergoing TKA and UKA. **(b)** Dumbbell-shaped tensile specimens. **(c)** The specimen is stretched until it breaks. **(d)** Stress-strain curves for tensile tests. **(e)** Fabrication of compression specimens. **(f)** Compression experiments with the Bose machine.

A total of 42 patients who underwent total knee arthroplasty (TKA) or unicompartmental knee arthroplasty (UKA) were included in this study, from whom 66 menisci were obtained. Specifically, 13 male patients provided 19 menisci, and 29 female patients provided 47 menisci (Figure 1a). Patients had a mean age of 69.6 ± 3.7 years. All patients provided written consent to allow the surgically excised meniscus tissue to be used for mechanical analysis. All procedures were approved by the Medical Ethics Committee of Beijing Luhe Hospital affiliated with Capital Medical University before the study started. Detailed information on the menisci, including patient age, sex, alignment parameters, symptom duration, contralateral knee status, and morphological classification, is provided in the Supplementary Materials.

Each meniscus was classified based on its morphological characteristics (Pauli et al., 2011): Level 0 - Normal; Level 1 - Marginal wear; Level 2 - Partial substance tear or wear; Level 3 - Near complete tear with minor tissue loss; Level 4 - Complete tear with significant tissue loss. The cohort included 13 Level 2, 30 Level 3, and 23 Level four menisci. All frozen samples were soaked in phosphate-buffered saline (PBS) solution 1 day before the experiment until thawed.

### 2.2 Tensile experiment

Based on preliminary experiments using pig meniscus, five rectangular and five dumbbell-shaped specimens were prepared. Each specimen was stretched to failure at a constant speed of 0.05 mm/s using a BOSE machine (BOSE 3330; BOSE Corporation, Eden Prairie, MN, USA). It was observed that the rectangular specimens failed near the grips, while 80% of the dumbbell-shaped specimens failed in the middle region. Therefore, for the actual experiments, the dumbbell-shaped specimens were used.

For the tensile tests, each of the 23 menisci was embedded and serially sectioned radially using a microtome to produce slices approximately 1 mm thick. To eliminate interference from superficial tissue, the outer 2–3 mm of both the superior and inferior surfaces of each meniscus were removed, retaining only the central portion for specimen preparation. On each slice, a custom-made dumbbell-shaped mold was used to excise samples along the circumferential direction, while avoiding areas adjacent to tears or regions of visible degeneration (Figure 1b). Due to varying degrees of damage among the harvested menisci, a total of 113 tensile test specimens were obtained from the 23 menisci (Table 1). The width and thickness of each specimen were measured using a digital caliper to ensure dimensional accuracy for subsequent mechanical testing. The specimens were held at both ends using a fixture and subjected to a constant strain rate of 0.05 mm/s until failure using a BOSE machine (BOSE 3330; BOSE Corporation, Eden Prairie, MN, USA) (Figure 1c). Throughout the experiment, phosphate-buffered saline (PBS) was regularly sprayed onto the specimen surface to minimize dehydration. After the experiment, force-displacement curves were obtained and converted into stress-strain curves to calculate the tensile modulus (Figure 1d). The engineering strain was computed as the change in gauge length divided by the original length, while engineering stress was calculated by dividing the measured force by the specimen’s original cross-sectional area.

**TABLE 1:** Number of samples for tensile testing.

Degeneration grade	Medial meniscus (n)	Lateral meniscus (n)
2	14	23
3	34	20
4	11	11

To calculate the elastic modulus, the nonlinear toe region, which reflects the uncrimping of collagen fibers, was excluded. The elastic modulus was defined as the slope of the linear portion of the stress-strain curve, determined by performing a least-squares linear regression on this region (Proctor et al., 1989). All calculations were performed using Origin (OriginLab Corporation, Northampton, MA, USA).

### 2.3 Compression experiment

For the compression tests, cylindrical specimens were prepared from 43 menisci using a custom-designed punch with a diameter of 6 mm (Figure 1e), avoiding areas with tears or visible degeneration to ensure tissue integrity. From each meniscus, as many specimens as possible were collected, depending on the available intact tissue. The upper and lower surfaces of each specimen were carefully trimmed to obtain flat, parallel faces, and the edges were removed to ensure uniform geometry. The final thickness of each specimen was approximately 4 mm. Due to the limited intact tissue in the harvested menisci caused by varying degrees of degeneration or damage, a total of 137 compression test specimens were obtained from the 43 menisci (Table 2). The thickness and dimensions of each specimen were measured using a vernier caliper to ensure dimensional accuracy for subsequent mechanical testing.The specimen was compressed using the BOSE machine (BOSE 3330; BOSE Corporation, Eden Prairie, MN, USA) at a rate of 1 mm/min in the vertical direction until a 30% strain was achieved (Figure 1f) (Freutel et al., 2014; Kolaczek et al., 2016; van Rossom et al., 2018). Throughout the experiment, phosphate-buffered saline (PBS) was regularly sprayed onto the specimen surface to minimize dehydration. After the experiment, force-displacement curves were obtained and converted into stress-strain curves to calculate the compressive modulus. The engineering strain was calculated as the change in specimen height divided by the original height, while engineering stress was calculated as the measured force divided by the original cross-sectional area.

**TABLE 2:** Number of samples for compression testing.

Degeneration grade	Medial meniscus (n)	Lateral meniscus (n)
2	0	28
3	15	52
4	21	21

To characterize the compressive stiffness, compressive moduli were calculated at specific strain levels of 10%, 20%, and 30%. For each target strain, a linear regression was performed over a small strain range (±2%) around the target point to determine the local slope of the stress-strain curve, which was taken as the compressive modulus at that strain. All calculations were performed using Origin (OriginLab Corporation, Northampton, MA, USA).

### 2.4 Inverse finite element method

Finite element models of the meniscus specimens were established using Abaqus (SIMULIA, Vélizy-Villacoublay, France) with dimensions corresponding to the actual experimental specimens, simulating both tensile and compression experiments.

The continuum mechanics was applied to characterize the strain-energy density function W using the three invariants of the Cauchy-Green deformation tensor. The strain-energy density function consists of two parts: the deviation term and the volume term, which are represented in polynomial form in Equation 1:

W=∑i,j=0NCijI1−3iI2−3j+∑k=1N1DkI3−122k, (1)

where the values of *i, j, k* can range from one to 3. $C_{ij}$ and $D_{k}$ represent material parameters, while $I_{1}$ 、 $I_{2}$ 、and $I_{3}$ denote the three tensor invariants.

Relevant studies have indicated that the human meniscus is composed of water (65%–72%), collagen (20%–25%), and proteoglycan (<1%) (Gee and Posner, 2021). These components form a dense extracellular matrix (ECM) populated with embedded cells, making the meniscus a structurally complex biphasic tissue that exhibits solid–fluid interactions. In its native physiological state, the meniscus exhibits poroviscoelastic behavior mainly due to fluid flow through its porous ECM (Travascio and Jackson, 2017). However, this study focuses on approximating the apparent elastic response of the solid matrix under quasi-static loading conditions. A simplified incompressible hyperelastic model was adopted to describe this short-term response. The relatively fast loading rates (tensile: 0.05 mm/s; compression: 1 mm/min) helped to reduce the time-dependent effects of fluid movement. As a result, the volume term is 0, i.e., $I_{3}=1$ . Therefore, Equation 1 simplifies to Equation 2:

$$W=\sum_{i,j=0}^{N}C_{ij}{\left(I_{1}-3\right)}^{i}{\left(I_{2}-3\right)}^{j},$$ (2)

The human meniscus tissue exhibits a nonlinear mechanical behavior, and the hyperelastic intrinsic relationship is able to take into account the property of the material to recover its original shape after large deformations, which is consistent with the actual situation of the meniscus tissue during human movement. During normal human movement such as walking, running, jumping, *etc.*, the meniscus is subjected to complex deformation, but it returns to its original shape at the end of the movement, and the Mooney - Rivlin model is able to describe this elastic recovery based on its strain energy function, and accurately simulate the deformation and recovery process of the meniscus under different stress states (Abraham et al., 2011b). The strain energy function of the two-parameter Mooney-Rivlin model is given by Equation 3 (Sun et al., 2019; Yamashita et al., 2023):

$$W_{M-R}=C_{10}\left(I_{1}-3\right){+C_{11}\left(I_{1}-3\right)\left(I_{2}-3\right)+C}_{01}\left(I_{2}-3\right).$$ (3)

For the approximately incompressible isotropic hyperelastic materials, the contribution of the higher order terms to the overall strain energy is relatively small in the case of small deformations, and at this time the $C_{11}$ term can be neglected, so that a simplified second order model can be used to describe the mechanical behaviour of the material, which can meet certain accuracy requirements and simplify the calculation process. The simplified Mooney-Rivlin model is given by Equation 4:

$$W_{M-R}=C_{10}\left(I_{1}-3\right){+C}_{01}\left(I_{2}-3\right).$$ (4)

In simulated tensile experiment, the bottom of the specimen was fully fixed, while the upper end was stretched along the Z-axis at a rate of 0.05 mm/s. In the compression experiment, the bottom of the specimen was fully fixed, and the upper surface was compressed along the Z-axis at a rate of 1 mm/min.

The residual sum of squares was chosen as the objective function for assessing the goodness of fit between experimental and simulated curves, calculated as Equation 5:

$$S=\sum {\left(y_{i}-\bar{y_{i}}\right)}^{2},$$ (5)

where *S* is the residual sum of squares, $y_{i}$ is the experimental data, and $\bar{y_{i}}$ is the simulated data. The set of results with the smallest residual sum of squares was chosen as the optimal solution.

The Non-Dominated Sorting Genetic Algorithm II (NSGA-II) was chosen as the optimization algorithm because of its ability to progressively approximate the optimal Pareto front, i.e., no one objective can be improved any further without making the other objectives worse. By maintaining a set of nondominated solutions, a balance can be found between these multiple objectives, providing a set of optimal trade-off solutions between the different objectives, instead of only one optimal solution as in the case of single-objective optimization algorithms (Zhang et al., 2022; Zhang et al., 2024).

Specific parameters were set as follows: Population Size is 52, Number of Generation is 100, Crossover Probability is 0.9, and Crossover Distribution Index is 20.

### 2.5 Statistical analysis

Factorial analysis of variance (ANOVA) was performed using SPSS statistical software to assess the differences in biomechanical properties between the medial and lateral menisci as well as degenerative grades of the menisci. The dependent variables assessed included tensile modulus and compression modulus. (Significance level α = 0.05).

Before conducting the ANOVA, we tested the assumptions of normality and homogeneity of variances. Normality was assessed using the Shapiro-Wilk test, and homogeneity of variances was evaluated using Levene’s test.

Post-hoc analyses were conducted using Tukey’s Honest Significant Difference (HSD) test to identify specific group differences when significant main effects or interactions were found. These *post hoc* tests were used to adjust for multiple comparisons and ensure a robust interpretation of the results.

## 3 Results

### 3.1 Tensile experiment

**FIGURE 2:**
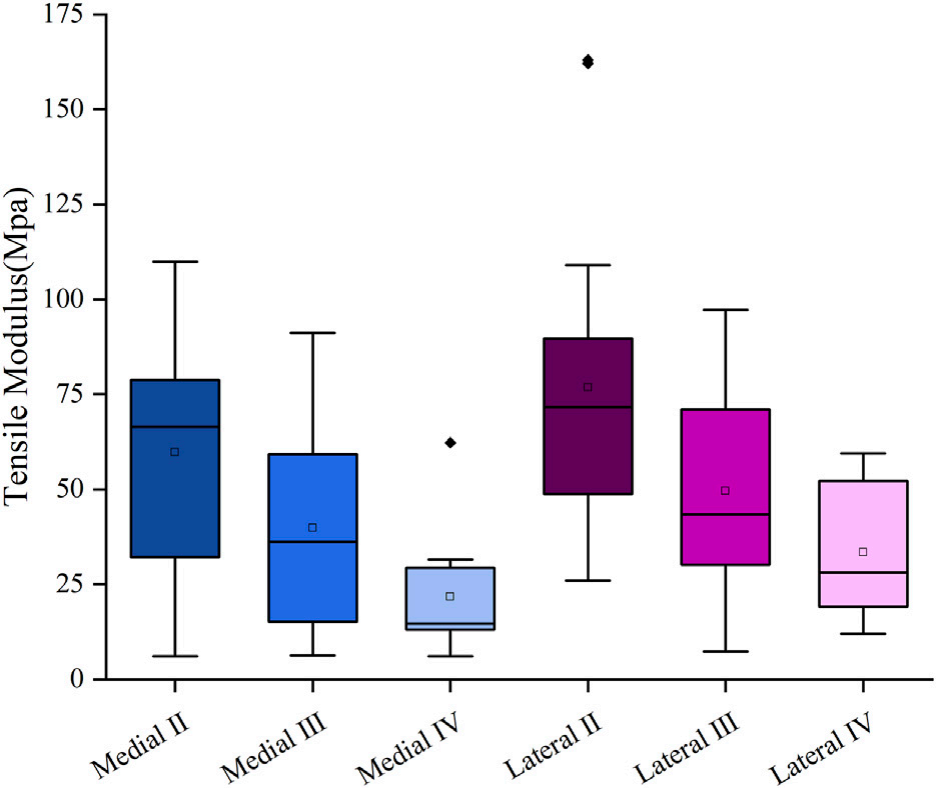
Tensile modulus at different meniscus degeneration levels.

The mean tensile modulus of the medial meniscus (n = 10) in patients with severe KOA was 41.2 ± 28.8 MPa and 57.9 ± 34.1 MPa in the lateral meniscus (n = 13). The tensile modulus gradually decreases as the meniscus degeneration level increases. The tensile modulus decreased sequentially by 33.38% and 45.4% for the medial meniscus, 32.1% and 32.5% for the lateral meniscus, respectively, compared with that of the graded two, three, and four menisci (Figure 2).

### 3.2 Compression experiment

**FIGURE 3:**
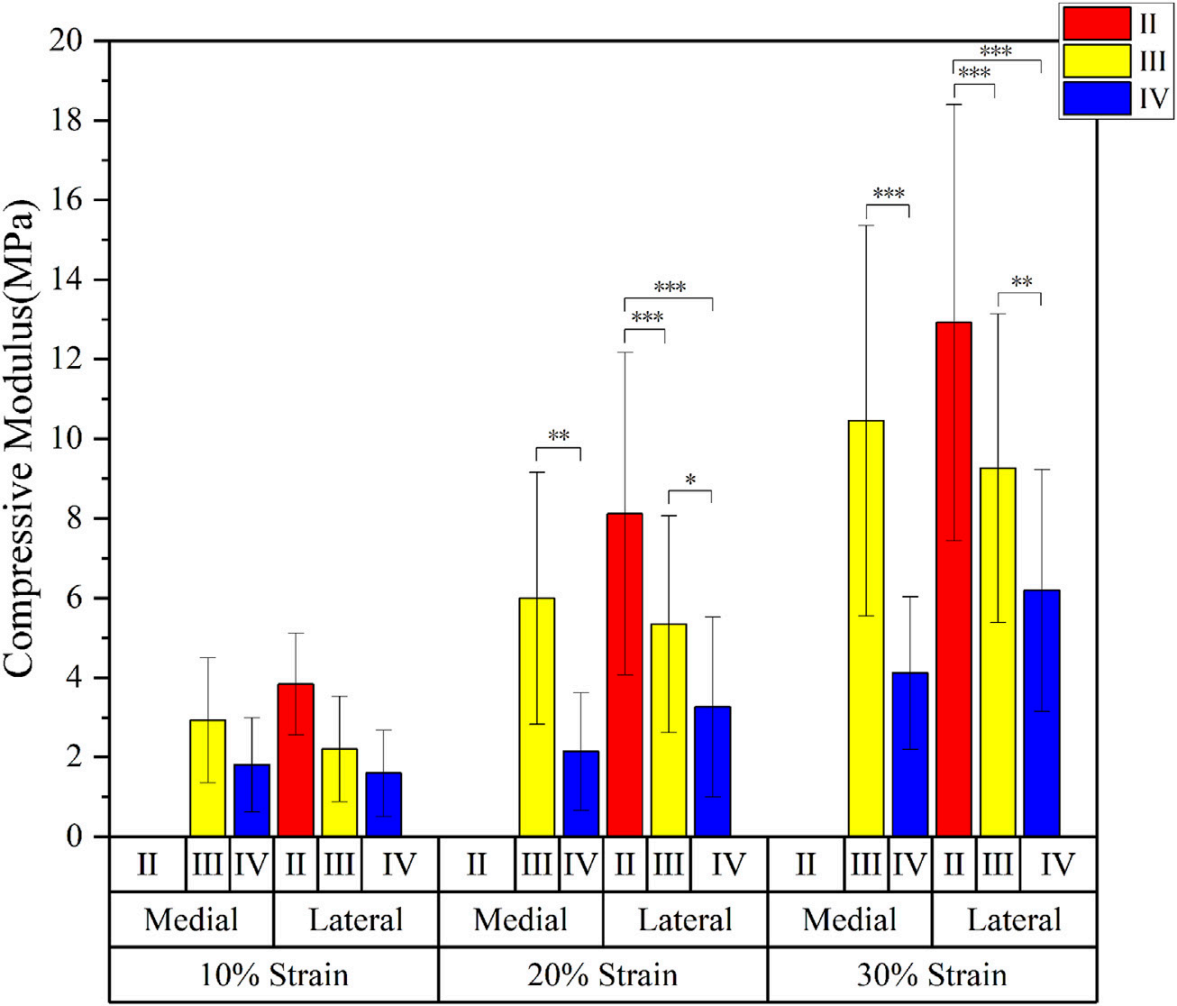
Mean and standard deviation of meniscus compression modulus at different strain levels.

The compressive modulus of the medial meniscus (n = 16) in patients with severe KOA was 2.3 ± 0.9 MPa, 3.5 ± 3.0 MPa, and 6.6 ± 4.7 MPa at 10%, 20%, and 30% strain levels, respectively; and 2.9 ± 1.2MPa, 6.4 ± 3.6MPa, and 12.5 ± 4.8 MPa in the lateral meniscus (n = 27). The compressive modulus increases as the strain level rises. The compression modulus tended to decrease gradually with the progression of meniscal degeneration in all three groups of strain levels (10%, 20%, and 30%) (Figure 3).

### 3.3 Inverse finite element method

**FIGURE 4:**
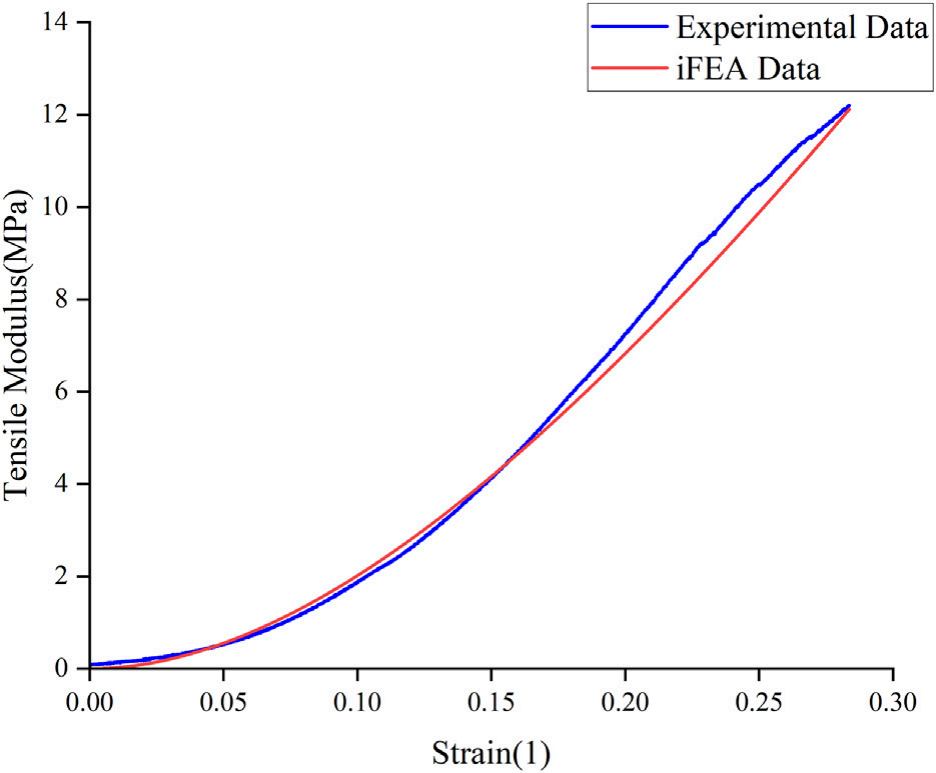
Experimental data curves and fitted curves from iFEA.

The Mooney-Rivlin constitutive model has two unknown parameters, C_10_ and C_01_. This study summarizes the optimization results of the biomechanical property models for different regions of the meniscus in severe KOA patients, obtained from the tensile and compression experiments, respectively (Table 3, 4). The goodness of fit (R^2^) was greater than 0.9 for all groups, and the model was considered to better characterize the biomechanical properties of the meniscus in patients with severe KOA. Figure 4 presents the agreement between the experimental stress-strain curves and the fitted curves obtained from the iFEA, showing the predictive capability of the Mooney-Rivlin model.

**TABLE 3:** Summary of mooney-rivlin model optimization results for tensile experiments.

Classification (number)	C_10_ (MPa)	C_01_ (MPa)	R^2^
Grade 2 medial(14)	40.49 ± 12.02	−40.49 ± 12.02	0.950 ± 0.027
Grade 2 lateral(23)	43.74 ± 13.56	−42.82 ± 12.64	0.933 ± 0.016
Grade 3 medial(34)	28.15 ± 6.47	−28.05 ± 6.29	0.955 ± 0.018
Grade 3 lateral(20)	33.62 ± 8.60	−33.62 ± 8.60	0.918 ± 0.048
Grade 4 medial(11)	11.82 ± 4.87	−10.87 ± 5.10	0.920 ± 0.026
Grade 4 lateral(11)	20.23 ± 6.28	−20.23 ± 6.28	0.945 ± 0.033

**TABLE 4:** Summary of mooney-rivlin model optimization results for compression experiments.

Classification (number)	C_10_ (MPa)	C_01_ (MPa)	R^2^
Grade 2 lateral(28)	6.27 ± 0.76	−6.27 ± 0.76	0.937 ± 0.050
Grade 3 medial(14)	4.03 ± 0.98	−4.03 ± 0.98	0.924 ± 0.025
Grade 3 lateral(53)	4.78 ± 0.80	−4.78 ± 0.80	0.919 ± 0.029
Grade 4 medial(23)	1.24 ± 0.58	−1.24 ± 0.58	0.965 ± 0.019
Grade 4 lateral(21)	2.84 ± 0.43	−2.84 ± 0.43	0.944 ± 0.037

## 4 Discussion

This study performed *ex vivo* measurements of the tensile and compressive modulus on meniscal tissue obtained from patients with severe KOA and assessed the effect of different factors of the meniscus (medial meniscus, lateral meniscus, and degree of degeneration) on the tensile and compressive modulus of the meniscus. By using the iFEA and experimental data, a constitutive model of the meniscus in severe KOA patients was developed.

### 4.1 Biomechanical properties of the meniscus in patients with severe KOA

We propose that the modulus difference between medial and lateral menisci is related to their microstructure. The meniscus comprises approximately 70% water and 30% organic matter, primarily collagen fibers (75% of organic content), glycosaminoglycans (GAGs), and minor components. Recent studies have indicated that the lateral meniscus has a significantly higher collagen content than the medial meniscus at various tissue depths (Danso et al., 2017). This higher collagen content may contribute to the superior mechanical properties of the lateral meniscus. The increased collagen content enhances the structural integrity and load-bearing capacity of the tissue, making the lateral meniscus better able to resist mechanical stresses and less prone to degeneration or tearing compared to the medial meniscus.

However, it is important to note that this conclusion is based on *in vitro* experiments. *In vivo*, the lateral meniscus exhibits greater mobility than the medial meniscus due to its less constrained anatomical attachments and more extensive range of motion during joint movement (Thompson et al., 1991; Fox et al., 2015). This increased mobility exposes the lateral meniscus to more variable loading patterns, which may influence its mechanical properties as an adaptive response. Thus, while our findings provide valuable insights into the structural basis of meniscal modulus differences, their direct extrapolation to *in vivo* conditions requires caution.​​

The study results from Fischenich et al. found that there was no statistical difference in the tensile moduli of the meniscus at different degeneration levels, which was mainly due to the small sample size (Fischenich et al., 2015). Warnecke et al. found that with the degeneration of the lateral meniscus, its tensile modulus showed a decreasing trend, although without a significant difference (Warnecke et al., 2020). However, the results of the present study show a negative correlation between the degeneration level of the meniscus in patients with severe KOA and both the tensile and compressive moduli. Bursac et al. found that the compressive performance of the meniscus is not related to collagen content but is significantly influenced by GAG content (Bursac et al., 2009). GAGs can control fluid flow, interact with other components such as collagen fibers, and maintain the structural integrity and stability of the meniscus. Therefore, a decrease in GAG content may lead to structural instability, reduced elasticity, and toughness of the meniscus, increased joint friction, and ultimately result in joint wear and degeneration.

### 4.2 Deviations between literature data and data from this study

Tissakht and Ahmed reported that the average tensile modulus of the healthy medial meniscus was 82.98 MPa, while that of the lateral meniscus was 111.66 MPa (Tissakht and Ahmed, 1995). In contrast, the current study found that the medial meniscus in patients with severe KOA had a significantly lower tensile modulus of 41.2 ± 28.8 MPa, and the lateral meniscus had a modulus of 57.9 ± 34.1 MPa. To statistically verify these differences, a one-sample t-test was performed comparing our experimental values with the literature-reported values. The results confirmed that the tensile modulus of both medial and lateral menisci in severe KOA patients was significantly reduced (p < 0.001 for both comparisons). This indicates a substantial mechanical deterioration in the menisci associated with severe KOA, underscoring the necessity of using degeneration-specific biomechanical parameters in modeling and simulations rather than relying solely on literature values derived from non-degenerated tissues.

Currently, in the literature, two models have been applied for the meniscus of patients with KOA in the finite element analysis: a linearly elastic material with an elastic modulus of 59 MPa (Yang et al., 2024; Park et al., 2019), and a linearly elastic, transversely isotropic material with a circumferential modulus of 120 MPa and an axial modulus of 5 MPa (Daszkiewicz and Łuczkiewicz, 2021). The inadequacy of the first model is that it treats the meniscus as a material with both tensile and compressive moduli of 59 MPa. However, through the mechanical tests in this study and data from other literature (Proctor et al., 1989; Orton et al., 2023; Tissakht and Ahmed, 1995), the difference between the tensile modulus and the compressive modulus is around two to three orders of magnitude. The problem with the second model is that it considers the tensile modulus in the circumferential direction of the meniscus as 120 MPa, assuming that the meniscus of KOA patients has a similar tensile modulus to that of healthy individuals (Lechner et al., 2000; Fischenich et al., 2015; Henderson et al., 2022). Therefore, both of these settings (Fischenich et al., 2015; Warnecke et al., 2020; Fischenich et al., 2017), which are commonly used today, differ from the actual situation.

### 4.3 Recommendations for data that can be used in modeling simulations

In view of the above analyses, this study proposes a hyperelastic model for meniscus material in patients with severe KOA. The Neo-Hookean hyperelasticity model was initially used to obtain optimization results for two parameters, C_10_ and D_1_, respectively. Each subgroup was validated with five sets of experimental data curves respectively, but the validation results obtained from this model were not satisfied. Then we chose the Mooney-Rivlin hyperelastic model to obtain optimization results for the parameters. After validation, we found that the goodness of fit for each group of curves was greater than 0.9, so we believe that this model for describing the meniscus material is reasonable. Due to the significant differences in biomechanical characteristics among different degeneration levels and portions (medial and lateral) of the meniscus, the results of this study provide the corresponding constitutive parameters for each group. In the future studies, when constructing finite element models, investigators can select appropriate material parameters based on the degeneration status of the patients' meniscus. For example, for the medial meniscus with a degeneration grade of 2, in the tensile experiment, the C_10_ value of the Mooney-Rivlin model is 40.49 ± 12.02 MPa, and the C_01_ value is −40.49 ± 12.02 MPa. In the compression experiment, for the lateral meniscus with a degeneration grade of 2, the C_10_ value is 6.27 ± 0.76 MPa, and the C_01_ value is −6.27 ± 0.76 MPa, *etc.* (Table 3, 4).

### 4.4 Estimation of mechanical properties in in-vivo conditions and characteristics and effectiveness of the model

In this study, iFEA was used to predict the biomechanical properties of the meniscus in patients with severe KOA. By combining experimental data with finite element simulation, the mechanical properties of the meniscus can be estimated more accurately. The constructed Mooney - Rivlin hyperelasticity models were able to fit the experimental data well, and the goodness-of-fit (R2) of the models were all greater than 0.9, which indicated that the models were able to effectively describe the mechanical behavior of meniscus in patients with severe KOA. In contrast to other studies (De Rosa et al., 2022; Seyfi et al., 2018; Freutel et al., 2015), this study gave corresponding model parameters for both medial and lateral human menisci of different grades. However, there are some limitations in this study. A further increase in the sample size would allow the mechanical characterisation of the anterior, middle and posterior meniscus, which is currently not statistically significant in this regard. At the same time, only the hyperelastic material model was considered in the iFEA, which inherently assumes an instantaneous elastic response and neglects time-dependent behaviors such as viscoelasticity and poroelasticity. The meniscus is a structurally complex tissue exhibiting anisotropy, viscoelasticity, and poroelastic effects due to fluid flow within its matrix (Seyfi et al., 2018; Freutel et al., 2015). Therefore, the use of a purely hyperelastic model limits the ability to fully capture the real physiological mechanical response of the tissue, especially under dynamic or long-duration loading conditions. Incorporating viscoelastic or visco-hyperelastic constitutive models in future studies would help to more accurately replicate the meniscus material behavior and improve the estimation of its mechanical properties *in vivo*.

## 5 Conclusion

For patients with severe KOA, the tensile and compressive moduli of the medial meniscus were lower than those of the lateral meniscus. Both the tensile and compressive moduli of menisci in patients with severe KOA decreased progressively with increasing meniscal degeneration level. Mechanical properties of meniscus in patients with severe KOA obtained in this study were significantly lower than the data from healthy individuals in the literature. An iFEA developed with the Mooney-Rivlin hyperelasticity model of meniscus was validated with tensile and compression experimental data of different degeneration levels of menisci. The goodness-of-fit values (R^2^) for all curves in iFEA model were greater than 0.9, showing promising application in predicting the biomechanical properties of the meniscus in patients with severe KOA.

Future research should incorporate larger sample sizes and explore additional material characteristics such as viscoelasticity to further refine meniscal models. These enhancements will improve the accuracy of *in vivo* estimations and provide valuable insights for knee osteoarthritis research and treatment strategies.

## Data Availability

The raw data supporting the conclusions of this article will be made available by the authors, without undue reservation.

## References

[B1] Abraham A. C. Edwards C. R. Odegard G. M. Donahue T. L. (2011a). Regional and fiber orientation dependent shear properties and anisotropy of bovine meniscus. J. Mech. Behav. Biomed. Mater. 4, 2024–2030. 10.1016/j.jmbbm.2011.06.022 22098902 PMC3222856

[B2] Abraham A. C. Moyer J. T. Villegas D. F. Odegard G. M. Haut Donahue T. L. (2011b). Hyperelastic properties of human meniscal attachments. J. Biomech. 44, 413–418. 10.1016/j.jbiomech.2010.10.001 20980006 PMC3022997

[B3] Andrews S. H. Rattner J. B. Abusara Z. Adesida A. Shrive N. G. Ronsky J. L. (2014). Tie-fibre structure and organization in the knee menisci. J. Anat. 224, 531–537. 10.1111/joa.12170 24617800 PMC3981495

[B4] Bursac P. Arnoczky S. York A. (2009). Dynamic compressive behavior of human meniscus correlates with its extra-cellular matrix composition. Biorheology 46, 227–237. 10.3233/bir-2009-0537 19581729

[B5] Chen H. Liu L. Zhang Y. (2023). Finite element analysis of the knee joint stress after partial meniscectomy for meniscus horizontal cleavage tears. BMC Musculoskelet. Disord. 24, 744. 10.1186/s12891-023-06868-y 37726679 PMC10508030

[B6] Danso E. K. Oinas J. M. T. Saarakkala S. Mikkonen S. TöYRäS J. Korhonen R. K. (2017). Structure-function relationships of human meniscus. J. Mech. Behav. Biomed. Mater 67, 51–60. 10.1016/j.jmbbm.2016.12.002 27987426

[B7] Daszkiewicz K. Łuczkiewicz P. (2021). Biomechanics of the medial meniscus in the osteoarthritic knee joint. PeerJ 9, e12509. 10.7717/peerj.12509 34900428 PMC8627128

[B8] DE Rosa M. Filippone G. Best T. M. Jackson A. R. Travascio F. (2022). Mechanical properties of meniscal circumferential fibers using an inverse finite element analysis approach. J. Mech. Behav. Biomed. Mater 126, 105073. 10.1016/j.jmbbm.2022.105073 34999488 PMC9162054

[B9] Dong Y. Yan Y. Zhou J. Zhou Q. Wei H. (2023). Evidence on risk factors for knee osteoarthritis in middle-older aged: a systematic review and Meta analysis. J. Orthop. Surg. Res. 18, 634. 10.1186/s13018-023-04089-6 37641050 PMC10464102

[B10] Fischenich K. M. Lewis J. Kindsfater K. A. Bailey T. S. Haut Donahue T. L. (2015). Effects of degeneration on the compressive and tensile properties of human meniscus. J. Biomech. 48, 1407–1411. 10.1016/j.jbiomech.2015.02.042 25770751

[B11] Fischenich K. M. Boncella K. Lewis J. T. Bailey T. S. Haut Donahue T. L. (2017). Dynamic compression of human and ovine meniscal tissue compared with a potential thermoplastic elastomer hydrogel replacement. J. Biomed. Mater Res. A 105, 2722–2728. 10.1002/jbm.a.36129 28556414 PMC5747566

[B12] Fox A. J. Wanivenhaus F. Burge A. J. Warren R. F. Rodeo S. A. (2015). The human meniscus: a review of anatomy, function, injury, and advances in treatment. Clin. Anat. 28, 269–287. 10.1002/ca.22456 25125315

[B13] Freutel M. Seitz A. M. Galbusera F. Bornstedt A. Rasche V. Knothe Tate M. L. (2014). Medial meniscal displacement and strain in three dimensions under compressive loads: MR assessment. J. Magn. Reson Imaging 40, 1181–1188. 10.1002/jmri.24461 24323799

[B14] Freutel M. Galbusera F. Ignatius A. DüRSELEN L. (2015). Material properties of individual menisci and their attachments obtained through inverse FE-analysis. J. Biomech. 48, 1343–1349. 10.1016/j.jbiomech.2015.03.014 25843259

[B15] Fukubayashi T. Kurosawa H. (1980). The contact area and pressure distribution pattern of the knee. A study of normal and osteoarthrotic knee joints. Acta Orthop. Scand. 51, 871–879. 10.3109/17453678008990887 6894212

[B16] Gao Y. Yin D. Tang M. Zhao B. (2023). A three-dimensional fractional visco-hyperelastic model for soft materials. J. Mech. Behav. Biomed. Mater 137, 105564. 10.1016/j.jmbbm.2022.105564 36395676

[B17] Gee S. M. Posner M. (2021). Meniscus anatomy and basic science. Sports Med. Arthrosc. Rev. 29, e18–e23. 10.1097/jsa.0000000000000327 34398117

[B18] Giorgino R. Albano D. Fusco S. Peretti G. M. Mangiavini L. Messina C. (2023). Knee osteoarthritis: epidemiology, pathogenesis, and mesenchymal stem cells: what else is new? An update. Int. J. Mol. Sci. 24, 6405. 10.3390/ijms24076405 37047377 PMC10094836

[B19] Henderson B. S. Cudworth K. F. Wale M. E. Siegel D. N. Lujan T. J. (2022). Tensile fatigue strength and endurance limit of human meniscus. J. Mech. Behav. Biomed. Mater 127, 105057. 10.1016/j.jmbbm.2021.105057 35091175 PMC9925119

[B20] Karahan M. Kocaoglu B. Cabukoglu C. Akgun U. Nuran R. (2010). Effect of partial medial meniscectomy on the proprioceptive function of the knee. Arch. Orthop. Trauma Surg. 130, 427–431. 10.1007/s00402-009-1018-2 20012072

[B21] Katsuragawa Y. Saitoh K. Tanaka N. Wake M. Ikeda Y. Furukawa H. (2010). Changes of human menisci in osteoarthritic knee joints. Osteoarthr. Cartil. 18, 1133–1143. 10.1016/j.joca.2010.05.017 20633672

[B22] Kolaczek S. Hewison C. Caterine S. Ragbar M. X. Getgood A. Gordon K. D. (2016). Analysis of 3D strain in the human medial meniscus. J. Mech. Behav. Biomed. Mater 63, 470–475. 10.1016/j.jmbbm.2016.06.001 27484043

[B23] Korhonen R. K. Wong M. Arokoski J. Lindgren R. Helminen H. J. Hunziker E. B. (2002). Importance of the superficial tissue layer for the indentation stiffness of articular cartilage. Med. Eng. Phys. 24, 99–108. 10.1016/s1350-4533(01)00123-0 11886828

[B24] Lechner K. Hull M. L. Howell S. M. (2000). Is the circumferential tensile modulus within a human medial meniscus affected by the test sample location and cross-sectional area? J. Orthop. Res. 18, 945–951. 10.1002/jor.1100180614 11192255

[B25] Lei F. Szeri A. Z. (2007). Inverse analysis of constitutive models: biological soft tissues. J. Biomech. 40, 936–940. 10.1016/j.jbiomech.2006.03.014 16730739

[B26] Leroux M. A. Setton L. A. (2002). Experimental and biphasic FEM determinations of the material properties and hydraulic permeability of the meniscus in tension. J. Biomech. Eng. 124, 315–321. 10.1115/1.1468868 12071267

[B27] Mahmoudian A. Lohmander L. S. Mobasheri A. Englund M. Luyten F. P. (2021). Early-stage symptomatic osteoarthritis of the knee - time for action. Nat. Rev. Rheumatol. 17, 621–632. 10.1038/s41584-021-00673-4 34465902

[B28] Maritz J. Agustoni G. Dragnevski K. Bordas S. P. A. Barrera O. (2021). The functionally grading elastic and viscoelastic properties of the body Region of the knee meniscus. Ann. Biomed. Eng. 49, 2421–2429. 10.1007/s10439-021-02792-1 34075449 PMC8455388

[B29] Markes A. R. Hodax J. D. Ma C. B. (2020). Meniscus form and function. Clin. Sports Med. 39, 1–12. 10.1016/j.csm.2019.08.007 31767101

[B30] Martin Seitz A. Galbusera F. Krais C. Ignatius A. DüRSELEN L. (2013). Stress-relaxation response of human menisci under confined compression conditions. J. Mech. Behav. Biomed. Mater 26, 68–80. 10.1016/j.jmbbm.2013.05.027 23811278

[B31] Michael J. W. SchlüTER-Brust K. U. Eysel P. (2010). The epidemiology, etiology, diagnosis, and treatment of osteoarthritis of the knee. Dtsch. Arztebl Int. 107, 152–162. 10.3238/arztebl.2010.0152 20305774 PMC2841860

[B32] Morejon A. Dalbo P. L. Best T. M. Jackson A. R. Travascio F. (2023). Tensile energy dissipation and mechanical properties of the knee meniscus: relationship with fiber orientation, tissue layer, and water content. Front. Bioeng. Biotechnol. 11, 1205512. 10.3389/fbioe.2023.1205512 37324417 PMC10264653

[B33] Moyer J. T. Priest R. Bouman T. Abraham A. C. Donahue T. L. (2013). Indentation properties and glycosaminoglycan content of human menisci in the deep zone. Acta Biomater. 9, 6624–6629. 10.1016/j.actbio.2012.12.033 23321302 PMC3628809

[B34] Orton K. Batchelor W. Ziebarth N. M. Best T. M. Travascio F. Jackson A. R. (2023). Biomechanical properties of porcine meniscus as determined *via* AFM: effect of region, compartment and anisotropy. PLoS One 18, e0280616. 10.1371/journal.pone.0280616 36662701 PMC9858324

[B35] Park S. Lee S. Yoon J. Chae S. W. (2019). Finite element analysis of knee and ankle joint during gait based on motion analysis. Med. Eng. Phys. 63, 33–41. 10.1016/j.medengphy.2018.11.003 30482441

[B36] Pauli C. Grogan S. P. Patil S. Otsuki S. Hasegawa A. Koziol J. (2011). Macroscopic and histopathologic analysis of human knee menisci in aging and osteoarthritis. Osteoarthr. Cartil. 19, 1132–1141. 10.1016/j.joca.2011.05.008 21683797 PMC3217905

[B37] Proctor C. S. Schmidt M. B. Whipple R. R. Kelly M. A. Mow V. C. (1989). Material properties of the normal medial bovine meniscus. J. Orthop. Res. 7, 771–782. 10.1002/jor.1100070602 2677284

[B38] Sandmann G. H. Eichhorn S. Vogt S. Adamczyk C. Aryee S. Hoberg M. (2009). Generation and characterization of a human acellular meniscus scaffold for tissue engineering. J. Biomed. Mater Res. A 91, 567–574. 10.1002/jbm.a.32269 18985757

[B39] Sandmann G. H. Adamczyk C. Grande Garcia E. Doebele S. Buettner A. Milz S. (2013). Biomechanical comparison of menisci from different species and artificial constructs. BMC Musculoskelet. Disord. 14, 324. 10.1186/1471-2474-14-324 24237933 PMC3840579

[B40] Saygi B. Yildirim Y. Berker N. Ofluoglu D. Karadag-Saygi E. Karahan M. (2005). Evaluation of the neurosensory function of the medial meniscus in humans. Arthroscopy 21, 1468–1472. 10.1016/j.arthro.2005.09.006 16376237

[B41] Seyfi B. Fatouraee N. Imeni M. (2018). Mechanical modeling and characterization of meniscus tissue using flat punch indentation and inverse finite element method. J. Mech. Behav. Biomed. Mater 77, 337–346. 10.1016/j.jmbbm.2017.09.023 28965040

[B42] Son M. Goodman S. B. Chen W. Hargreaves B. A. Gold G. E. Levenston M. E. (2013). Regional variation in T1ρ and T2 times in osteoarthritic human menisci: correlation with mechanical properties and matrix composition. Osteoarthr. Cartil. 21, 796–805. 10.1016/j.joca.2013.03.002 23499673 PMC3909565

[B43] Sun Y. Chen L. Yick K. L. Yu W. Lau N. Jiao W. (2019). Optimization method for the determination of Mooney-Rivlin material coefficients of the human breasts *in-vivo* using static and dynamic finite element models. J. Mech. Behav. Biomed. Mater 90, 615–625. 10.1016/j.jmbbm.2018.11.016 30500699

[B44] Sweigart M. A. Zhu C. F. Burt D. M. Deholl P. D. Agrawal C. M. Clanton T. O. (2004). Intraspecies and interspecies comparison of the compressive properties of the medial meniscus. Ann. Biomed. Eng. 32, 1569–1579. 10.1114/b:abme.0000049040.70767.5c 15636116

[B45] Thompson W. O. Thaete F. L. Fu F. H. Dye S. F. (1991). Tibial meniscal dynamics using three-dimensional reconstruction of magnetic resonance images. Am. J. Sports Med. 19, 210–216. 10.1177/036354659101900302 1867329

[B46] Tissakht M. Ahmed A. M. (1995). Tensile stress-strain characteristics of the human meniscal material. J. Biomech. 28, 411–422. 10.1016/0021-9290(94)00081-e 7738050

[B47] Travascio F. Jackson A. R. (2017). The nutrition of the human meniscus: a computational analysis investigating the effect of vascular recession on tissue homeostasis. J. Biomech. 61, 151–159. 10.1016/j.jbiomech.2017.07.019 28778387

[B48] Vaienti E. Scita G. Ceccarelli F. Pogliacomi F. (2017). Understanding the human knee and its relationship to total knee replacement. Acta Biomed. 88, 6–16. 10.23750/abm.v88i2-S.6507 28657560 PMC6178997

[B49] VAN Rossom S. Smith C. R. Thelen D. G. Vanwanseele B. VAN Assche D. Jonkers I. (2018). Knee joint loading in healthy adults during functional Exercises: implications for Rehabilitation guidelines. J. Orthop. Sports Phys. Ther. 48, 162–173. 10.2519/jospt.2018.7459 29308697

[B50] Walker P. S. Erkman M. J. (1975). The role of the menisci in force transmission across the knee. Clin. Orthop. Relat. Res. 109, 184–192. 10.1097/00003086-197506000-00027 1173360

[B51] Warnecke D. Balko J. Haas J. Bieger R. Leucht F. Wolf N. (2020). Degeneration alters the biomechanical properties and structural composition of lateral human menisci. Osteoarthr. Cartil. 28, 1482–1491. 10.1016/j.joca.2020.07.004 32739340

[B52] Yamashita Y. Uematsu H. Tanoue S. (2023). Calculation of strain energy density function using ogden model and mooney-rivlin model based on biaxial elongation experiments of silicone rubber. Polym. (Basel) 15, 2266. 10.3390/polym15102266 37242841 PMC10221263

[B53] Yang Y. Guo Y. Wang C. Zhang X. Zhang K. Ji B. (2024). Finite element analysis of sagittal angles of unicompartmental knee arthroplasty. Clin. Biomech. (Bristol, Avon) 114, 106232. 10.1016/j.clinbiomech.2024.106232 38547571

[B54] Zhang J. Yao Y. Sun W. Tang L. Li X. Lin H. (2022). Application of the non-dominated sorting genetic algorithm II in multi-objective optimization of orally disintegrating tablet formulation. AAPS PharmSciTech 23, 224. 10.1208/s12249-022-02379-6 35962205

[B55] Zhang F. Wen B. Niu D. Li A. Guo B. (2024). Optimized design of low-carbon mix ratio for non-dominated sorting genetic algorithm II concrete based on genetic Algorithm-improved back propagation. Mater. (Basel) 17, 4077. 10.3390/ma17164077 39203254 PMC11356359

